# Does the nitrogen single-breath washout test contribute to detecting pulmonary involvement in rheumatoid arthritis? A pilot study

**DOI:** 10.1186/s13104-019-4767-1

**Published:** 2019-11-07

**Authors:** Elizabeth Jauhar Cardoso Bessa, Felipe de Miranda Carbonieri Ribeiro, Geraldo da Rocha Castelar Pinheiro, Agnaldo José Lopes

**Affiliations:** 1 Postgraduate Programme in Medical Sciences, School of Medical Sciences, State University of Rio de Janeiro (UERJ), Boulevard 28 de Setembro, 77, Vila Isabel, Rio de Janeiro, 20551-030 Brazil; 2 Clinica Felippe Mattoso/Hospital Samaritano, Rua Marechal Niemeyer, 25, Botafogo, Rio de Janeiro, 22251-060 Brazil; 3 Rehabilitation Sciences Postgraduate Programme, Augusto Motta University Centre (UNISUAM), Rua Dona Isabel, 94, Bonsucesso, Rio de Janeiro, 21032-060 Brazil; 4 Rua Araguaia, 1266, Bloco 1/405, Freguesia/Jacarepaguá, Rio de Janeiro, RJ 22745-271 Brazil

**Keywords:** Rheumatoid arthritis, Pulmonary function test, Small airway disease, Computed tomography

## Abstract

**Objective:**

There has been growing interest in studying small airway disease through measures of ventilation distribution, thanks to the resurgence of the nitrogen single-breath washout (N_2_SBW) test. Therefore, this study evaluated the contribution of the N_2_SBW test to the detection of pulmonary involvement in patients with rheumatoid arthritis (RA).

**Results:**

Twenty-one patients with RA underwent clinical evaluation, pulmonary function tests (PFTs), including the N2_S_BW test, and computed tomography (CT). The main tomographic findings were air trapping and bronchiectasis (57.1% and 23.8% of cases, respectively). According to the phase III slope of the N_2_SBW (phase III slope), 11 and 10 patients had values < 120% predicted and > 120% predicted, respectively. Five patients with limited involvement on CT had a phase III slope > 120%. The residual volume/total lung capacity ratio was significantly different between patients with phase III slopes < 120% and > 120% (*P *= 0.024). Additionally, rheumatoid factor positivity was higher in patients with a phase III slope > 120% (*P *= 0.021). In patients with RA and airway disease on CT, the N_2_SBW test detects inhomogeneity in the ventilation distribution in approximately half of the cases, even in those with normal conventional PFT results.

## Introduction

In rheumatoid arthritis (RA), extra-articular disease occurs in approximately 50% of patients, and the lung is a common site of involvement [1]. Computed tomography (CT) and pulmonary function tests (PFTs), including spirometry and diffusing capacity for carbon monoxide (DLco), are the pillars of clinical assessment of patients with pulmonary involvement in RA. Spirometry is an effort-dependent test that only provides a single global measure and, thus, is not sensitive enough to detect regional disease; it is also poor for assessing early onset of lung disease. The same occurs with the ability of CT to detect small airway disease (SAD) and evaluate individuals with early pulmonary fibrosis [2].

In recent years, there has been growing interest in the study of SAD through measures of ventilation distribution inhomogeneity. This interest is due to the resumption of the nitrogen single-breath washout (N_2_SBW) test, a direct result of the technological advancement of the tools used to measure it [3]. Because many patients with RA may have normal conventional PFTs in the presence of radiological changes, the need to study the N_2_SBW test in this population arises. Thus, we evaluated the contribution of the N_2_SBW test to detecting pulmonary involvement in patients with RA and the association of this test with clinical findings, conventional PFTs and CT abnormalities.

## Main text

### Methods

This cross-sectional study evaluated 21 consecutive patients aged > 18 years regularly seen at the Department of Rheumatology of the Piquet Carneiro Polyclinic, State University of Rio de Janeiro, Rio de Janeiro, Brazil, and who met the diagnostic criteria of RA [4]. Smokers or former smokers who smoked > 10 pack-years, patients with asthma or chronic obstructive pulmonary disease (COPD), and those with respiratory infection within the last 3 weeks were excluded. The protocol was approved by the local ethics committee, and all patients signed an informed consent form.

All participants were evaluated by the Clinical Disease Activity Index (CDAI), which is calculated from the following four variables: the number of tender joints, the number of swollen joints, patient global assessment of disease activity using a visual analogue scale, and physician global assessment of disease activity [5]. The CDAI can vary between 0 and 76, and a higher value indicates higher disease activity. Serum samples were obtained to measure rheumatoid factor (RF) and anti-cyclic citrullinated peptide (anti-CCP) antibodies. The cut-off values for RF positivity (latex agglutination; Bioclin Wama) and anti-CCP (immunofluorometric assay; Thermo Fisher ImmunoCAP Assays) were ≥ 8 IU/mL and > 10 EliA U/mL, respectively.

Spirometry, body plethysmography, and DLco measurements were performed on an HDpft 3000 instrument (nSpire Health, Inc., Longmont, CO, USA), using reference values for the evaluated parameters [6–8]. In addition, we also performed the N_2_SBW test on the same instrument. Briefly, the individuals exhaled until reaching the residual volume (RV) and then inhaled 100% O_2_ until the total lung capacity (TLC) was reached. They then exhaled slowly to the RV at a flow rate of approximately 0.3 to 0.5 L/s. The exhaled N_2_ concentration was reported through a device located at the airway opening [3]. In interpreting the N_2_SBW test, we used the phase III slope, which is the change in the N_2_ concentration between 25 and 75% of the expired volume; this variable was analysed relative to the predicted value [9].

CT exams were performed on a 64-channel helical CT scanner (Brilliance 40, Philips Medical Systems, Cleveland, OH, USA). The voltage was 120 kV. Each image acquisition consisted of a block with 146–247 2-mm-thick cross-sections and a 2-mm distance between the cross-sections obtained during inspiratory and expiratory apnoea. The images were represented by a square matrix (512 rows and 512 columns). The gantry did not incline, and iodinated contrast medium was not used. CT images were analysed by two independent readers with 5 and 21 years of experience in pulmonary imaging who were blinded to the clinical data and PFT results. Disagreements were resolved by consensus after joint reassessment of the CT scans. The chest CT result was classified as limited (< 20%) or extensive (> 20%) according to the extent of pulmonary parenchymal involvement [10]. Air trapping (AT) was defined on end-expiration CT scans as parenchymal areas with a less-than-normal increase in attenuation and a lack of volume reduction [11]. AT was subdivided into mild (< 25%), moderate (25–50%), and severe (> 50%) based on a subjective evaluation of the total lung volume of air trapped in the lung [12]. Bronchiectasis criteria included bronchial dilation with respect to the accompanying pulmonary artery, lack of tapering of the bronchi, and identification of bronchi within 1 cm of the pleural surface [11, 13]. Bronchial wall thickening was assessed subjectively (peribronchial cuffing), and uncertain cases were assessed by measuring the ratio between the short-axis internal bronchial diameter and the short-axis diameter of the accompanying pulmonary artery [14, 15].

Data analysis was performed using SAS 6.11 (SAS Institute, Inc., Cary, NC, USA). The phase III slope was dichotomized into < 120% or > 120% of predicted, which was considered the cut-off point for the upper limit to define abnormality [16]. Comparison of the variables between the two groups of patients subdivided according to the phase III slope was evaluated by the Mann–Whitney test for numeric variables and the Fisher exact test for categorical variables. The phase III slope was compared between the three groups of patients subdivided according to the degree of AT using Kruskal–Wallis ANOVA. The results are expressed as the median and interquartile ranges or as frequencies (percentages); a statistical significance level of *P *< 0.05 was adopted.

### Results

In the evaluated sample, which consisted of 21 women, only 2 patients (9.52%) had a history of smoking. Eleven patients had respiratory symptoms. The most commonly used medications were methotrexate (n = 15), leflunomide (n = 7), and prednisone (n = 2).

Regarding the CT exams, 7 (33.3%) individuals had extensive involvement on CT, and 3 (14.3%) had normal CT scans. The tomographic findings were distributed as follows: 12 (57.1%) patients with AT; 5 (23.8%) with bronchiectasis; 3 (14.3%) with localized subpleural reticulation; 3 (14.3%) with bubbles; 3 (14.3%) with atelectasis; 2 (9.52%) with emphysema; and 1 (4.76%) with non-extensive usual interstitial pneumonia. Among 21 CT exams, 9 (42.9%) showed no AT, 8 (38.1%) showed mild AT, and 4 (19%) showed moderate AT.

**Fig. 1:**
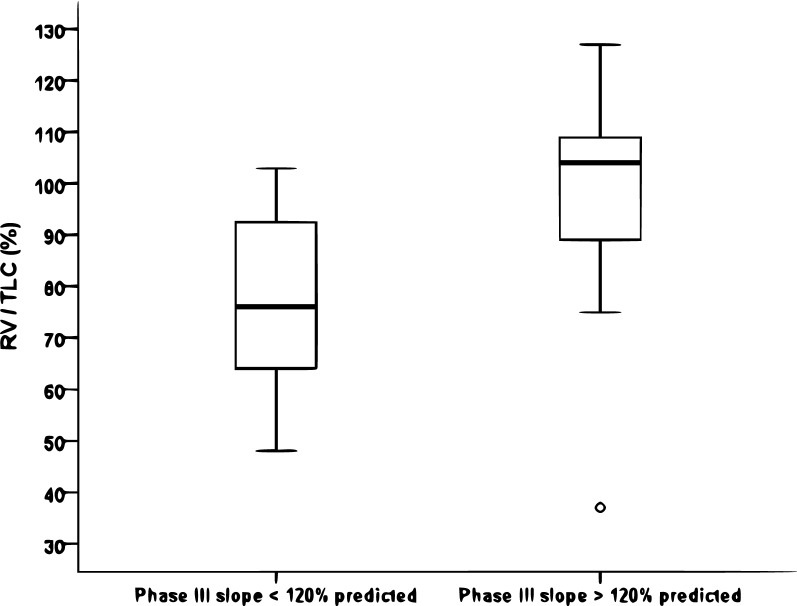
Box plots (median, 1st and 3rd quartiles, minimum and maximum) of the residual volume/total lung capacity (RV/TLC) ratio according to the phase III slope of the nitrogen single-breath washout (phase III slope). A significant difference was found between patients with phase III slope < 120% and patients with phase III slope > 120% (*P *= 0.024)

**Fig. 2:**
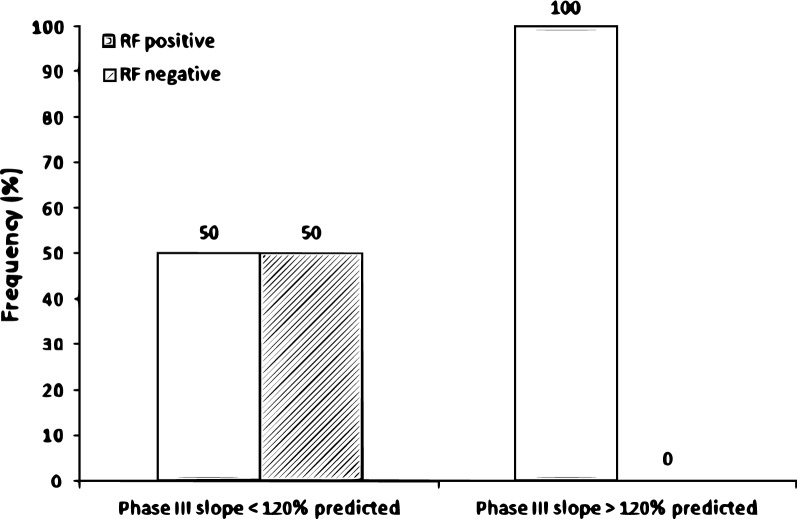
Positive and negative rheumatoid factor (RF) frequencies according to the phase III slope of the nitrogen single-breath washout (phase III slope). A significant difference was found between groups of patients (*P *= 0.021)

Regarding conventional PFTs, the forced vital capacity (FVC) and DLco were > 80% predicted in two and four cases, respectively; no patient showed a forced expiratory volume in 1 s (FEV_1_)/FVC < 70%. Regarding the N_2_SBW test, the median phase III slope was 106 (82–268)% predicted, with 11 and 10 patients presenting values < 120% and > 120% predicted, respectively. The median percentages of the predicted value of these two groups were 84 (64–93) and 268 (198–465) (*P *= 0.0001), respectively. Five (35.7%) patients with limited involvement on CT had a phase III slope > 120% (2 of whom had completely normal CT scans), while 5 (71.4%) patients with extensive involvement on CT had a phase III slope > 120%. We observed no significant difference in the phase III slope between the subgroups of patients with no AT, mild AT, and moderate AT [188 (93–279) vs. 86 (68–141) vs. 147 (61–344)% predicted, respectively; P = 0.25]. The comparisons of the clinical findings, serology, pulmonary function and disease extent on CT according to the phase III slope are shown in Table 1 and Figs. 1, 2.

**Table 1:** Clinical data, serology, lung function and computed tomography of the study population and of the groups according to phase III slope

Variable	Total sample (n = 21)	Phase III slope < 120% (n = 11)	Phase III slope > 120% (n = 10)	*P*-value between groups
Clinical data				
Age (years)	54 (42–67.5)	48 (34–56)	66.5 (51.5–71.3)	*0.020*
BMI (kg/m^2^)	27.6 (23.9–29.5)	25 (23.1–29.4)	28 (26.3–29.8)	0.16
Time from onset of symptoms to diagnosis (months)	12 (6–24)	9 (6–18)	18 (26.3–27)	0.80
Time since diagnosis (months)	204 (114–252)	168 (108–240)	216 (26.3–276)	0.31
CDAI (score)	13 (9.50–18.5)	13 (10–19)	17 (26.3–19.8)	0.80
Serology*				
RF positivity (n, %)	14 (73.7)	5 (50)	9 (100)	*0.021*
RF (IU/mL)	128 (5–256)	23.9 (5–320)	256 (112–256)	0.10
Anti-CPP positivity (n, %)	17 (89.5)	8 (80%)	9 (100%)	0.75
Anti-CCP (IU/mL)	126 (42–340)	104.5 (31.8–347.4)	175 (51.5–340)	0.90
Lung function				
FVC (% predicted)	102 (91–105)	102 (93–105)	101 (85.3–107)	0.94
FEV_1_ (% predicted)	95 (90.5–107.5)	104 (91–109)	92.5 (86–102)	0.17
FEV_1_/FVC (%)	86 (77.5–88.5)	86 (85–88)	80 (74.8–89.5)	0.36
TLC (% predicted)	92 (85.5–97)	89 (83–96)	96.5 (89.8–101.5)	0.13
RV (% predicted)	78 (52–105)	68 (54–86)	99.5 (50–114)	0.097
RV/TLC (%)	91 (66.5–105)	76 (63–94)	104 (85.5–113)	*0.024*
DLco (% predicted)	89 (82–100.5)	89 (85–96)	89.5 (72.5–106)	0.86
CT				
CT with involvement > 20% (n, %)	7 (33)	2 (18.2)	5 (50)	0.14

Significant *P* values are given in italic (*P* < 0.05)

*Phase III* phase III slope of the nitrogen single-breath washout, *BMI* body mass index, *CDAI* Clinical Disease Activity Index, *RF* rheumatoid factor, *anti-CCP* anti-cyclic citrullinated peptide antibodies, *FVC* forced vital capacity, *FEV*_*1*_ forced expiratory volume in 1 s, *TLC* total lung capacity, *RV* residual volume, *DLco* diffusing capacity for carbon monoxide, *CT* computed tomography

Results expressed as the median (interquartile range) or number (%). *n = 19

### Discussion

In the present study, we were careful to eliminate the impact of smoking on pulmonary function deterioration and SAD development; therefore, we evaluated only individuals with a smoking status < 10 pack-years without a history of asthma or COPD. The evaluated sample consisted predominantly of patients with bronchial disease or no pulmonary involvement, as observed by CT. This may be partially explained by the fact that smoking is currently linked to interstitial lung disease development in RA [17]. The main findings of the present study were that in patients with RA, the N_2_SBW test may be altered even in individuals with limited pulmonary parenchymal involvement, including subjects with normal CT. In these patients, a relationship was found between the phase III slope and the RV/TLC; the latter is an index used as a screening tool for SAD. In addition, the phase III slope was higher in RF-positive patients.

Several studies on SAD in patients with RA have produced controversial results [18–21], which can be explained mainly by differences in the diagnostic tools used. Most of these studies used forced expiratory flow during the middle half of the FVC (FEF_25–75%_) to diagnose SAD. However, changes in FEF_25–75%_ are nonspecific and show an unacceptably large number of false-negative and false-positive results. Moreover, the reduction in FEF_25–75%_ values is a result of changes in the resistance and susceptibility of the surrounding lung parenchyma, rather than obstruction of a specific airway segment [22].

In recent years, the resurgence of the N_2_SBW test with modern equipment has enabled a more reliable assessment of ventilation distribution inhomogeneity and SAD. Further evidence of this test’s association with small airway inflammation was identified from examinations of bronchial biopsies and bronchoalveolar lavage specimens [23]. In this scenario, we demonstrated high phase III slope values in 10 of 21 non-smoking patients with RA, some of whom had normal CT. To our knowledge, only one other study has used the N_2_SBW test for SAD assessment in patients with RA [19]. Contrary to our results, that study observed an elevation in the phase III slope in only 16% of its sample. A possible explanation for the discrepancy between the results of the two studies may be the evolution of the technological device, which allowed a more reliable analysis of the phase III slope.

In the present study, patients with higher phase III slope values showed higher RV/TLC values. This finding reinforces the use of RV/TLC as an indirect marker to assess SAD [24]. The latter is characterized by a progressive increase in resistance as the lung empties and regional inhomogeneity of the flow and time constants, in addition to premature closure of the airways signalled by the increased RV/TLC [25]. In addition, we observed an association between phase III slope elevation and RF positivity. Using FEF_25–75%_ as a marker of SAD in RA, a recent study showed no association of this parameter with RF or anti-CCP (which are the biomarkers most used in the diagnosis and prognosis of RA in clinical practice) [20]. Interestingly, Park et al. [26] demonstrated an association between anti-CCP positivity and small airway abnormalities evaluated by CT. However, it is important to note that smoking itself increases protein citrullination, leading to higher anti-CCP production in individuals with RA [27].

Notably, the main tomographic finding observed in our sample was the presence of AT, which may be at least partly explained by the predominance of patients with airway disease. This finding is in line with those of Perez et al. [19] and Lin et al. [28], who also observed a high AT prevalence in RA patients. However, similar to Perez et al. [19], we failed to demonstrate any association between the phase III slope and the presence of AT on CT, indicating that imaging findings and functional results do not always reflect the same pathological abnormalities that occur in RA. Recent advances in imaging research, especially xenon contrast-enhanced CT and hyperpolarized gas magnetic resonance imaging, may provide new insights into the dynamic nature of ventilation heterogeneity and SAD and thus improve correlations between imaging findings and pulmonary function [29].

In conclusion, our preliminary findings suggest that in a sample of patients with RA and a predominance of airway disease on CT, the N_2_SBW test is able to detect inhomogeneity in ventilation distribution in approximately half of the cases. In addition, there is a relationship between this abnormality and increased RV/TLC and RF positivity.

## Limitations

The present study involved a small number of subjects and only women; therefore, a prospective study with a greater number of RA subjects who can be compared with a control group is necessary to generalize our results. Moreover, we did not calculate the inter-rater agreement for the visual evaluations of the CT scans.

## Data Availability

All the data supporting the results are provided in the manuscript.

## Abbreviations

Anti-CCP: anti-cyclic citrullinated peptide
AT: air trapping
CDAI: Clinical Disease Activity Index
COPD: chronic obstructive pulmonary disease
CT: computed tomography
DLco: diffusing capacity for carbon monoxide
FEF_25–75%_: forced expiratory flow during the middle half of the forced vital capacity
FEV_1_: forced expiratory volume in 1 s
N_2_SBW: nitrogen single-breath washout
PFT: pulmonary function test
phase III slope: phase III slope of the nitrogen single-breath washout
RA: rheumatoid arthritis
RF: rheumatoid factor
RV: residual
SAD: small airway disease
TLC: total lung capacity

## References

[1] Yunt ZX, Solomon JJ (2015). Lung disease in rheumatoid arthritis. Rheum Dis Clin N Am.

[2] Gochuico BR, Avila NA, Chow CK, Novero LJ, Wu HP, Ren P (2008). Progressive preclinical interstitial lung disease in rheumatoid arthritis. Arch Intern Med.

[3] Robinson PD, Latzin P, Verbanck S, Hall GL, Horsley A, Gappa M (2013). Consensus statement for inert gas washout measurement using multiple- and single-breath tests. Eur Respir J.

[4] Aletaha D, Neogi T, Silman AJ, Funovits J, Felson DT, Bingham CO (2010). 2010 rheumatoid arthritis classification criteria: an American College of Rheumatology/European League Against Rheumatism collaborative initiative. Ann Rheum Dis.

[5] Aletaha D, Smolen J (2005). The Simplified Disease Activity Index (SDAI) and the Clinical Disease Activity Index (CDAI): a review of their usefulness and validity in rheumatoid arthritis. Clin Exp Rheumatol.

[6] Pereira CAC, Sato T, Rodrigues SC (2007). New reference values for forced spirometry in white adults in Brazil. J Bras Pneumol.

[7] Neder JA, Andreoni S, Castelo-Filho A, Nery LE (1999). Reference values for lung function tests. I. Static volumes. Braz J Med Biol Res.

[8] Neder JA, Andreoni S, Peres C, Nery LE (1999). Reference values for lung function tests. III. Carbon monoxide diffusing capacity (transfer factor). Braz J Med Biol Res.

[9] Teculescu DB, Damel MC, Costantino E, Buhler O, Bohadana AB, Marchand M (1996). Computerized single-breath nitrogen washout: predicted values in a rural French community. Lung.

[10] Kelly CA, Saravanan V, Nisar M, Arthanari S, Woodhead FA, Price-Forbes AN (2014). Rheumatoid arthritis-related interstitial lung disease: associations, prognostic factors and physiological and radiological characteristics: a large multicentre UK study. Rheumatology.

[11] Hansell DM, Bankier AA, MacMahon H, McLoud TC, Müller NL, Remy J (2008). Fleischner Society: glossary of terms for thoracic imaging. Radiology.

[12] Miller WT, Chatzkel J, Hewitt MG (2014). Expiratory air trapping on thoracic computed tomography: a diagnostic subclassification. Ann Am Thorac Soc.

[13] Naidich DP, McCauley DI, Khouri NF, Stitik FP, Siegelman SS (1982). Computed tomography of bronchiectasis. J Comput Assist Tomogr.

[14] Winningham PJ, Martínez-Jiménez S, Rosado-de-Christenson ML, Betancourt SL, Restrepo CS, Eraso A (2017). Bronchiolitis: a practical approach for the general radiologist. Radiographics.

[15] Silva CI, Colby TV, Müller NL (2004). Asthma and associated conditions: high-resolution CT and pathologic findings. Am J Roentgenol.

[16] Lopes AJ, Marinho CL, Alves UD, Gonçalves CEA, Silva PO, Botelho EC (2017). Relationship between ventilation heterogeneity and exercise intolerance in adults with sickle cell anemia. Braz J Med Biol Res.

[17] Johnson C (2017). Recent advances in the pathogenesis, prediction, and management of rheumatoid arthritis-associated interstitial lung disease. Curr Opin Rheumatol.

[18] Cortet B, Perez T, Roux N, Flipo RM, Duquesnoy B, Delcambre B (1997). Pulmonary function tests and high resolution computed tomography of the lungs in patients with rheumatoid arthritis. Ann Rheum Dis.

[19] Perez T, Remy-Jardin M, Cortet B (1998). Airways involvement in rheumatoid arthritis: clinical, functional, and HRCT findings. Am J Respir Crit Care Med.

[20] Mori S, Koga Y, Sugimoto M (2011). Small airway obstruction in patients with rheumatoid arthritis. Mod Rheumatol.

[21] Avnon LS, Manzur F, Bolotin A, Heimer D, Flusser D, Buskila D (2009). Pulmonary functions testing in patients with rheumatoid arthritis. Isr Med Assoc J.

[22] Piorunek T, Kostrzewska M, Stelmach-Mardas M, Mardas M, Michalak S, Goździk-Spychalska J (2017). Small airway obstruction in chronic obstructive pulmonary disease: potential parameters for early detection. Adv Exp Med Biol.

[23] McNulty W, Usmani OS (2014). Techniques of assessing small airways dysfunction. Eur Clin Respir J.

[24] Cottini M, Lombardi C, Micheletto C (2015). Small airway dysfunction and bronchial asthma control: the state of the art. Asthma Res Pract.

[25] Stewart JI, Criner GJ (2013). The small airways in chronic obstructive pulmonary disease: pathology and effects on disease progression and survival. Curr Opin Pulm Med.

[26] Park WH, Kim SS, Shim SC, Song ST, Jung SS, Kim JH (2016). Visual assessment of chest computed tomography findings in anti-cyclic citrullinated peptide antibody positive rheumatoid arthritis: is it associated with airway abnormalities?. Lung.

[27] Klareskog L, Stolt P, Lundberg K, Källberg H, Bengtsson C, Grunewald J (2006). A new model for an etiology of rheumatoid arthritis: smoking may trigger HLA-DR (shared epitope)-restricted immune reactions to autoantigens modified by citrullination. Arthritis Rheum.

[28] Lin E, Limper AH, Moua T (2018). Obliterative bronchiolitis associated with rheumatoid arthritis: analysis of a single-center case series. BMC Pulm Med.

[29] Bommart S, Marin G, Bourdin A, Molinari N, Klein F, Hayot M (2014). Relationship between CT air trapping criteria and lung function in small airway impairment quantification. BMC Pulm Med.

